# Draft Genome Sequence of *Pseudomonas aeruginosa* Strain Hex1T Isolated from Soils Contaminated with Used Lubricating Oil in Argentina

**DOI:** 10.1128/genomeA.01473-16

**Published:** 2017-01-12

**Authors:** Adela M. Luján, Sofía Feliziani, Andrea M. Smania

**Affiliations:** CIQUIBIC, Departamento de Química Biológica, Facultad de Ciencias Químicas, CONICET, Universidad Nacional de Córdoba, Córdoba, Argentina

## Abstract

*Pseudomonas aeruginosa* Hex1T was isolated from soils contaminated with used lubricating oil from a garage in Córdoba, Argentina. This strain is capable of utilizing this pollutant as the sole carbon and energy source. Here, we present the 6.9-Mb draft genome sequence of Hex1T, which contains many heavy metal-resistance genes.

## GENOME ANNOUNCEMENT

*Pseudomonas aeruginosa* is a Gram-negative bacterium thoroughly distributed in different environments such as water, soil, and plants. It has an extraordinary genetic and metabolic versatility that allows it to use distinctive compounds as sources of carbon and energy. The genome of *P. aeruginosa* is one of the biggest in the bacterial world, which contributes to its high versatility and competitiveness for adapting to different environments (1).

Among these environments, contaminated sites have received particular interest because of the possibility of bioremediation by microorganisms capable of degrading environmental pollutants. In particular, *P. aeruginosa* strain Hex1T, isolated from used motor oil-contaminated soils from Córdoba, Argentina (2), is able to grow in this pollutant as its sole carbon and energy source. Used motor oil contains heavy polycyclic aromatic hydrocarbons (PAHs) and high levels of metals (3). Therefore, the genome sequencing of strain Hex1T could give useful information about its biodegradation and toxic metal-resistance mechanisms.

Whole-genome sequencing was performed using a paired-end (PE) 2 × 100 bp library on an Illumina MiSeq (INDEAR Genome Sequencing facility, Argentina). The 9,930,896 obtained Illumina reads were processed and assembled using the A5 assembly pipeline (version A5-miseq 20,150,522), according to the workflow described by Tritt et al. (4). The assembly resulted in 51 contigs (minimum, 376 bp; maximum, 714,006 bp; *N*_50_, 301,903 bp). The final assembly contained 6,981,653 bp, with a G+C content of 66% and a median coverage of 75×.

The genome annotation was performed by submitting the sequences to the Rapid Annotations using Subsystems Technology (RAST) server (5). A total of 6,605 coding sequences (CDSs) were predicted.

According to the proposed PAHs degradation mechanisms (6), several putative PHAs catabolic enzymes were found in the Hex1T genome, such as mono and dioxygenases, dehydrogenases, and the cytochrome P450. In addition, Hex1T presented numerous (15) genes involved in bacterial resistance to arsenic (arsenic resistance protein ArsH, arsenical-resistance protein [ACR3], arsenical pump-driving ATPase, arsenate reductases, and transcriptional regulator ArsR), lead (lead transporting ATPases), mercury (mercury resistance operon regulatory protein, mercury resistance protein MerT, periplasmic mercury[+2] binding protein, and mercury ion reductase), copper (multicopper oxidase, copper resistance protein, CopG protein, copper translocating P-type ATPase, and copper resistance protein CopC) and chrome (superoxidase dismutase SodM-like protein ChrF, rhodanase-like protein ChrE, chromate transport protein ChrA). Notably, these genes are present in only a few strains of *P. aeruginosa*.

Information from whole-genome sequencing here obtained indicates that, in addition to various catabolic abilities, strain Hex1T could have unique resistance to heavy metals. These results will help to reveal the genes coding for enzymes supporting the capability of *P. aeruginosa* Hex1T to grow and degrade used lubricating oil.

### Accession number(s).

This whole-genome shotgun project has been deposited at DDBJ/ENA/GenBank under the accession no. LNIV00000000. The version described in this paper is version LNIV02000000.
